# A synchronous papillary and follicular thyroid carcinoma presenting as a large toxic nodule in a female adolescent

**DOI:** 10.1186/s13633-020-00084-4

**Published:** 2020-07-21

**Authors:** Joke Van Vlaenderen, Karl Logghe, Eva Schiettecatte, Hubert Vermeersch, Wouter Huvenne, Kathleen De Waele, Hanne Van Beveren, Jo Van Dorpe, David Creytens, Jean De Schepper

**Affiliations:** 1 Department of Pediatric Endocrinology, University Hospital Ghent, Corneel Heymanslaan 10, 9000 Ghent, Belgium; 2 Department of Pediatrics, AZ Delta, Roeselare, Brugsesteenweg 90, 8800 Roeselare, Belgium; 3 Department of Radiology, University Hospital Ghent, Corneel Heymanslaan 10, 9000 Ghent, Belgium; 4 Department of Head and Neck Surgery, University Hospital Ghent, Corneel Heymanslaan 10, 9000 Ghent, Belgium; 5 Department of Pathology, University Hospital Ghent, Corneel Heymanslaan 10, 9000 Ghent, Belgium; 6 Department of Pediatric Endocrinology, University Hospital Brussels, Laarbeeklaan 101, 1090 Brussels, Belgium

**Keywords:** Hyperthyroidism, Toxic nodule, Follicular thyroid carcinoma, Papillary thyroid carcinoma, Children, Pediatric

## Abstract

**Case presentation:**

We report for the first time a synchronous papillary and follicular thyroid carcinoma in a 12-year-old girl presenting with a large (5 cm diameter) left thyroid nodule, an increased left and right upper pole technetium tracer uptake at scintigraphy and hyperthyroidism. The uptake at the right lobe was explained by the crossing of the left nodule to the right site of the neck at Computed Tomography (CT) scanning.

**Background:**

Although thyroid nodules are less common in children than in adults, there is more vigilance required in children because of the higher risk of malignancy. According to literature, about 5% of the thyroid nodules in adults are malignant versus 20–26% in children. The characteristics of 9 other pediatric cases with a differentiated thyroid carcinoma presenting with a toxic nodule, which have been reported during the last 20 years, are summarized. A nodular size of more than 3.5 cm and female predominance was a common finding.

**Conclusions:**

The presence of hyperthyroidism in association with a hyperfunctioning thyroid nodule does not rule out thyroid cancer and warrants careful evaluation, even in the absence of cervical lymph node invasion.

## Background

Solitary thyroid nodules are rare in childhood in comparison with adulthood, but have a higher risk of malignancy [1]. Up to 26% of the thyroid nodules in children were found to be malignant [1–3]. Most differentiated thyroid cancers (DTC) in children are papillary carcinoma, which only very rarely (over) produce thyroid hormones, causing subclinical or overt hyperthyroidism [4]. In a recent pediatric cohort study of thyroid nodules, hyperthyroidism was found in only 5% [5]. The finding of a hyperfunctioning or hot nodule on scintigraphy in the context of hyperthyroidism, especially in the absence of enlarged cervical lymph nodes, is usually reassuring, as in most cases a benign follicular adenoma is diagnosed [5–7].

We additionally reviewed the previously reported DTC in children and adolescents with a hyperfunctioning thyroid nodule associated with hyperthyroidism, also called toxic nodule, to look for common characteristics and potential risk factors for malignancy. A literature search on Pubmed and Web of Science was performed in the English literature in a period from January 2009, when most laboratories were using a third generation TSH assay, until December 2019. The search terms used were “hyperthyroidism and thyroid carcinoma”, “toxic nodule”, “hyperfunctioning nodule and thyroid carcinoma” in conjunction with either “children” or “pediatric”. Inclusion criteria were detailed case descriptions of children aged below 16 years old with a clinical and biological hyperthyroidism and an underlying differentiated thyroid carcinoma documented at histological examination. Finally, 8 articles describing 9 cases were selected. Clinical characteristics, biological, imaging and histological results and initial treatment of these 9 pediatric patients are summarized in Table 1.

**Table 1:** **characteristics of 9 pediatric cases of a DTC reported during the last 20 years**

	Sex	Age	Complaints	TSH miU/L (ref)	fT3 pmol/L (ref)	fT4 pmol/l (ref)	Antibodies	US dimensions	US structure	Scintigraphy Activity in surrounding tissue	FNAC	Histology
**Mircescu 2000** [8]	F	11 y	Painless cervical mass right sided Tremor	↓ 0.03	5.7 (1.0–2.8)	> 75 (8–18)	Undetectable	45x37x28mm	Numerous cystic lesions	Suppresed activity	Not mentioned	PTC
**Tfayli 2010** [9]	F	11 y	Heavy menses Fatigue Right thyroid mass	Undetectable	5.9 (1.9–3.2)	14.7 (9.4–23.7)	Undetectable	30–35 mm	Large nonhomogenous nodule in right lobe	Remaining weak activity	Not diagnostic	PTC
**Damle 2011** [10]	M	2 m	Right sided neck swelling since birth Clinical features of thyrotoxicosis	Thyrotoxicosis	Thyrotoxicosis	Thyrotoxicosis	Not mentioned	34x22x20 mm	Nodule	Suppressed activity	Moderately cellular smear with clusters and sheets of epithelial cells	PTC
	F	7 y	Palpitations and tremor Right nodule	0.01 (0.5–4.5)	7.4 (1.0–3.0)	27.9 (5.8–15.4)	–	50 × 50 mm	–	Suppressed activity	Epithelial cell clusters with nuclear overlapping,	Follicular variant of PTC
**Gabalec 2013** [11]	F	15 y	Thyroid nodule self-palpation Sweating Insomnia	0.01 (0.3–5.6)		30.6 (7–15.9)	Undetectable	35x23x45mm	Asymmetrical, enlarged left lobe completely filled with heterogeneous well demarcated nodule	Suppressed activity	Suspicious	Follicular variant of PTC
**Ruggeri 2013** [12]	F	15 y	Painless mass Fatigue Weight loss Palpitations	0.001 (0.27–4.2)	7.7 (3.0–6.8)	25.9 (11.6–21.9)	Undetectable	35x30x21mm	Increase in size, intense intranodular bloodflow, isoechoic, nonhomogenous, regular margins, peripheral halo	Suppressed activity	–	Follicular variant of PTC
**Rees 2015** [13]	F	16 y	Left thyroid mass. Weight loss, tremor, frequent bowel movement, hair loss. Feeling tearful and anxious	0.03 (0.53–3.59)	14.3 (3.5–7.7)	39.4 (12–20.6)	Undetectable	40 × 25 mm	Hyperechoic, hypervascular nodule	Suppressed activity		Follicular variant of PTC
**Blackburn, 2018** [14]	F	12 y	Right-sided neck swelling, increasing in size over the previous four weeks	↓ < 0.03 3	9.1 (3.6–6.4)	10.1 (9–19)	Undetectable	21 × 17 × 17 mm	Heterogeneous highly vascular mass	Not mentioned	–	FTC
**Dy, 2018** [15]	F	14 y	Left-sided palpable thyroid lesion, increasing sweats, tremors and tachycardia	0.02 (0.4–5.6)	–	18.0 (11.6–19.3)	Not mentioned	34 × 21 × 29 mm	Hypoechoic, heterogeneous and hypervascular	Suppressed activity	Benign	FTC

*Abbreviations: ref, reference values; M, male; F, female; m, month; y, year; US, ultrasound; PTC, Papillary Thyroid Carcinoma; FTC, Follicular Thyroid Carcinoma*

### Overview of previously reported case reports of DTC in children with a toxic nodule [8–15]

All described patients were female except one. Age at presentation varied between a minimum age of 2 months and a maximum age of 16 years (median 11 years). The severity of the hyperthyroidism is not always mentioned, but FT4 concentrations up to 3 times the upper limit with less elevated FT3 concentrations were reported. Right as well as left sided localisation of the nodule was seen, while the longest diameter of the nodule ranged from 35 to 50 mm. Suppressed activity in surrounding tissue at scintigraphic evaluation was noted in 8 cases. In four cases a follicular variant of a papillary thyroid carcinoma was found, while in the others either a papillary thyroid carcinoma or a follicular carcinoma was present (Table 1).

## Case presentation

In a 12-year-old female, presenting with a painless neck swelling since one month, a 5 cm long, non-tender but firm nodule in the left thyroid lobe was detected at physical examination. There was no palpable cervical lymphadenopathy. Slight tachycardia (pulse rate 95/min), but no exophthalmos was present. The patient reported intermittent sore throat, increasing nervousness and a 2 kg weight loss in the last several months. There was no history of radiation exposure. Her mother had recently undergone surgery for a multi-nodular goitre. There was no family history of cancer or intestinal polyps.

**Fig. 1:**
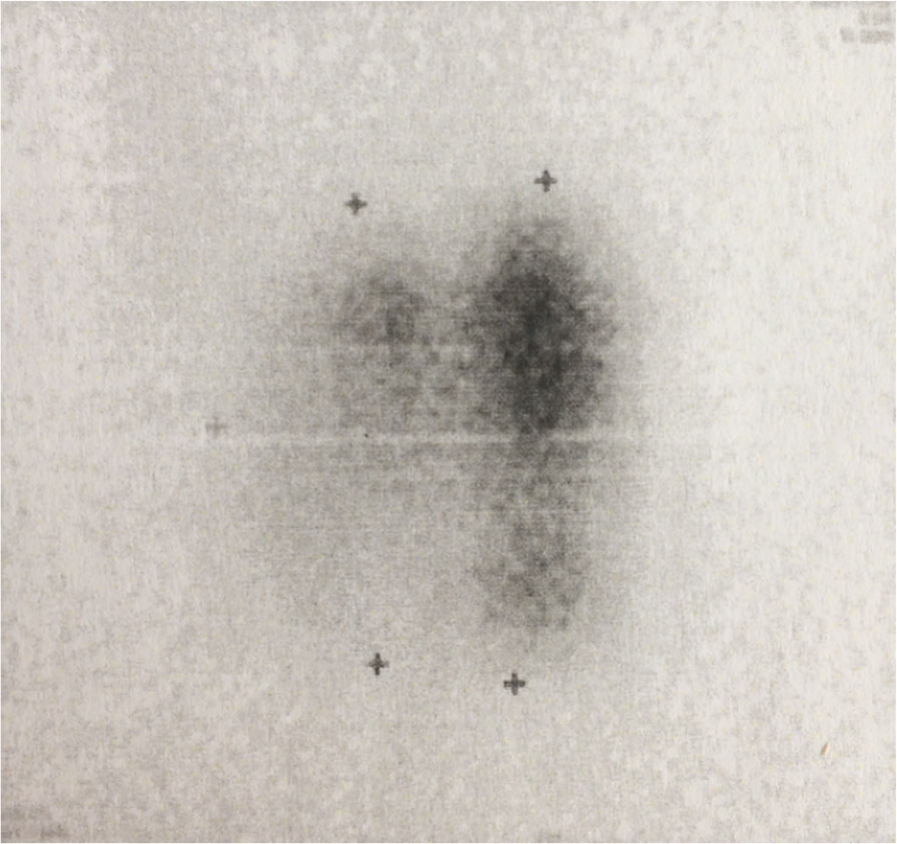
Scintigraphic image showing a global but heterogeneous hyperfunctioning thyroid gland with excessive uptake at upper left lobe and upper right lobe

**Fig. 2:**
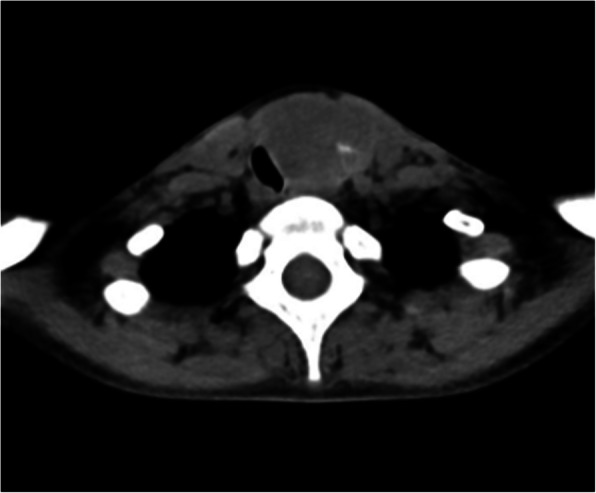
CT image showing a right tracheal deviation by a left thyroid mass

**Fig. 3:**
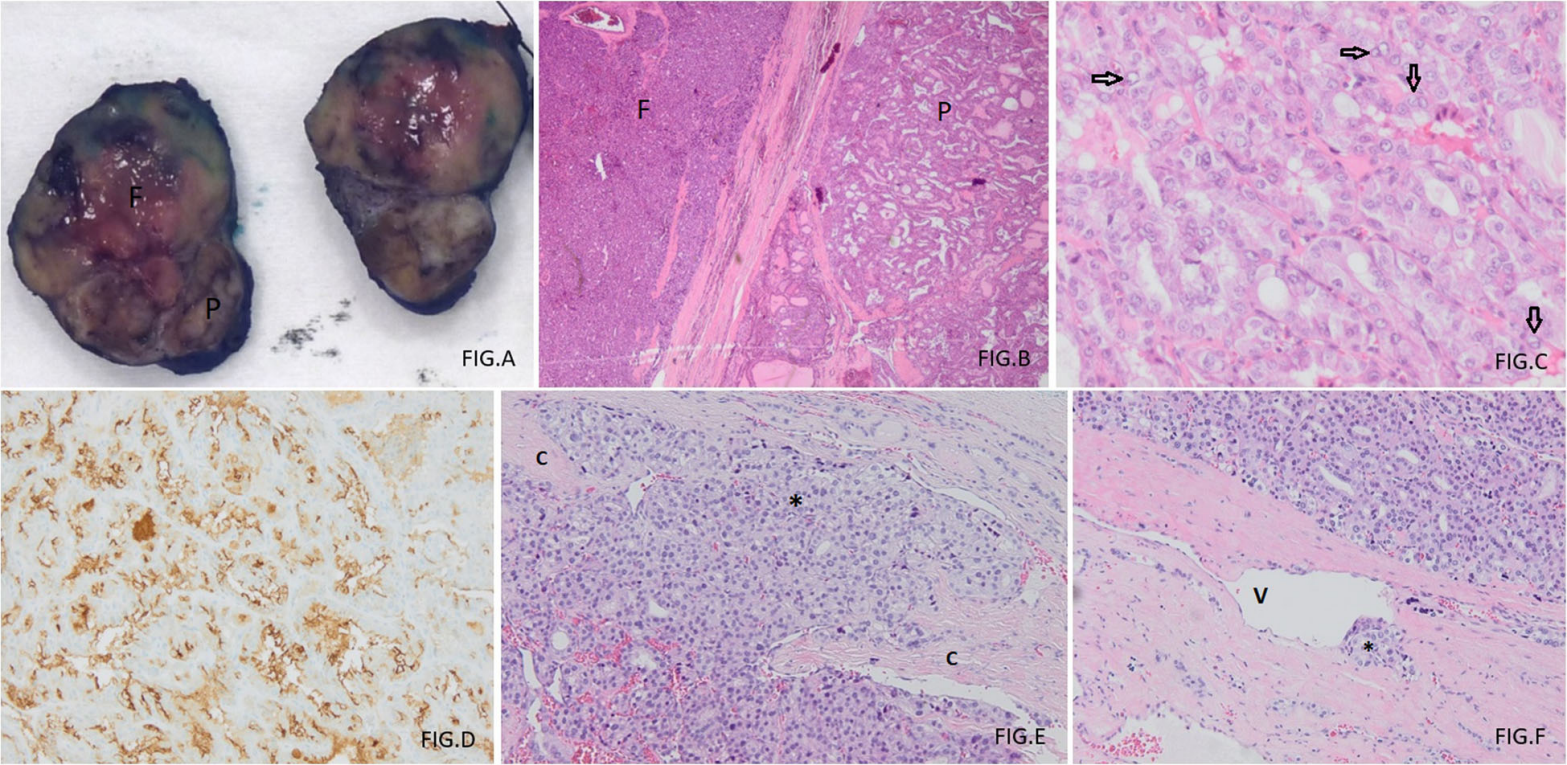
**a** Gross pathology (sections). Two separated neoplastic tumors in the left thyroid lobe: follicular thyroid carcinoma (F) (at the top) and papillary thyroid carcinoma (P) (at the bottom). **b** Two separated morphologically distinct neoplastic tumors in the left thyroid lobe: minimally invasive follicular thyroid carcinoma (F) (to the left) and papillary thyroid carcinoma (P) (to the right) (Hematoxylin and Eosin, original magnification 40x). **c** Cytonuclear features of papillary thyroid carcinoma, including nuclear overlapping, nuclear grooves (↓) and nuclear clearing (→) (Hematoxylin and Eosin, original magnification 200x). **d** Aberrant strong apical membranous (brown coloured) HBME expression in the papillary thyroid carcinoma (original magnification 200x). **e** Capsular invasion (C) in the follicular thyroid carcinoma (*) (Hematoxylin and Eosin, original magnification 100x). **f** Angioinvasive growth (V) in the follicular thyroid carcinoma (*) (Hematoxylin and Eosin, original magnification 100x)

Thyroid function tests showed an elevated FT4 (51 pmol/L or 39.6 pg/mL) and thyroglobulin concentration (435 μg/L) and a decreased TSH (< 0.005 mU/L) concentration.^1^ Serum anti-thyroglobulin, anti-thyroidperoxidase and anti-TSH receptor antibodies were undetectable. Doppler ultrasound showed a normal right lobe as well as a sharp defined hypervascular solid multilobular mass (longest diameter 53 mm) with cystic components with microcalcifications in the left lobe. A ^99m^Technetium scintigraphy (Fig. 1) showed a global, but heterogeneous hyperfunctioning thyroid gland with excessive uptake at the upper left lobe and upper right lobe. A right tracheal deviation by the left thyroid mass, but no cervical or mediastinal lymphnodes or lung masses, was seen on the CT scan of neck and thorax (Fig. 2). Subsequently, a large toxic adenoma crossing the midline was diagnosed, explaining the tracer uptake in the right upper pole region. Fine needle biopsy was refused by the patient and a left thyroidectomy was proposed. The patient underwent a left hemithyroidectomy after 2 months of methimazole therapy, which resulted in normalized thyroid function in one month’s time. Histological examination of the left lobe showed two separated morphologically distinct neoplastic lesions arising in what appeared to be a multinodular goitre. The largest lesion (measuring 2.9 cm) showed a follicular growth pattern with cytonuclear atypia, multiple foci of intracapsular vascular invasion and some foci of capsular invasion. Separated from this lesion by a couple of millimetres, a second neoplastic lesion was seen. This was non-encapsulated and infiltrative and had a papillary and follicular growth pattern with cytonuclear features of a papillary thyroid carcinoma (nuclear clearing, nuclear inclusions and grooves). The non-encapsulated, infiltrative neoplastic lesion with papillary architecture and cytomorphology showed aberrant apical and strong HBME immunohistochemical staining, whereas the adjacent lesion with follicular growth pattern was completely negative for HBME. Therefore, based on the morphology and immunohistochemistry, a diagnosis of a synchronous minimally invasive follicular thyroid carcinoma (with multifocal capsular invasion and angioinvasion) and a non-encapsulated, infiltrative (classical type) papillary thyroid carcinoma of the left lobe was made (Fig. 3). Consequently, total thyroidectomy was performed.

Genetic testing on the surgical specimen was negative for *BRAF* and *NRAS* mutations and *RET/PTC* rearrangements. Results of *TSHR* gene, *GNAS* gene and *PTEN* gene analysis were normal.

## Discussion

Hyperfunctioning nodules at thyroid scintigraphy, also called hot nodules, can present with or without hyperthyroidism. In the latter case, these nodules are also described as toxic nodules in literature. In the previously reported nine pediatric cases of DTC with associated hyperthyroidism and a hot nodule at scintigraphy, follicular carcinomas, papillary carcinomas as well as follicular variants of papillary thyroid carcinoma were diagnosed. We report for the first time a synchronous papillary-follicular thyroid carcinoma in female adolescent presenting with a toxic nodule. In our case as well as in the previously reported pediatric cases, the nodules were found to be greater than 3 cm in diameter, suggesting that clinical hyperthyroidism does not appear until the nodule is at least 3 cm in diameter. When comparing with non-hyperfunctioning nodules, thyroid nodules in hyperthyroid adolescents were found to have more compressive signs and a greater nodule size, and are mostly diagnosed as follicular adenomas (toxic adenoma) [5, 16].

The major goal of the diagnostic evaluation of thyroid nodules is to differentiate thyroid cancers, especially aggressive lesions, from benign adenomas. In the initial work-up of a thyroid nodular lesion, thyroid function tests are usually performed. The American Thyroid Association (ATA) Taskforce recommends that patients who have a thyroid nodule larger than 1 to 1.5 cm in any dimension, should have a serum thyrotropin (TSH) measurement [17]. If hyperthyroidism is associated with a nodule on ultrasound, a scintiscan is the next logical step to document the hyperfunctioning of the nodule, especially when thyroid stimulating immunoglobulines are absent. In toxic adenoma, the typical scintigraphic finding is a hot pattern in the nodule with the remnant thyroid tissue showing a severely decreased or absent uptake [18]. In our case no complete suppression was found, while in the other pediatric cases both complete and incomplete scintigraphic suppression patterns were reported. An incomplete suppression pattern was seen as a risk factor for DTC by Niedziela et al. [19] in his series of 31 children with a hyperfunctioning nodule.

The prevalence of malignancy in a hot nodule in adults has been estimated at 3.1% [20]. Histological outcome studies in children with a toxic nodule are very limited. No malignancy was detected in 6 Italian hyperthyroid pediatric patients with a solitary toxic nodule at surgery [5]. In an American study of 4 children with a hot or warm nodule and persisting T3 hyperthyroidism, no malignancy was found after partial thyroidectomy [18], while in another study of 2 hyperthyroid adolescents a follicular carcinoma was found in one female [21]. However, in a more recent study of 15 Polish children with hyperthyroidism and a hyperfunctioning nodule at scintigraphy, a DCT was diagnosed in 2 children after surgery [22]. In none of the reported adult or pediatric cases a simultaneous papillary and follicular carcinoma in a hot nodule was described. The simultaneous occurrence of different types of thyroid cancer in a single patient is very rare. Although there are noticeable reports about synchronous papillary cancer, the reports of simultaneous papillary and follicular cancer are actually rare [23]. This simultaneous thyroid tumor presentation has been described as coincidental in the literature as no common gene mutation for the pathogenesis of the different tumor types of the thyroid has been found. Mixed tumors however can occur as part of familial cancer syndromes. Cowden’s syndrome was excluded in our patient by genetic analysis, while no signs of Carney’s complex were present. Fine needle aspiration (FNA) biopsy is considered to be the most accurate procedure to identify malignant nodules, but is generally not advised in hot nodules. First, their larger size might easily result in false negative results. Second, hot nodules have lower likelihood of finding malignancy compared to cold nodules and third, there are histological difficulties in differentiating follicular adenoma from follicular thyroid carcinoma.

Contrary to guidelines for adults, the current recommendation for treatment of hyperfunctioning nodules in children is surgical resection rather than radio iodine treatment [11, 24]. Surgery is even more preferred when there are signs of compression, which is a common finding in hyperfunctioning nodules since they are larger in most cases than non-functional nodules [25]. Because of the absence of clinical (male gender, age < 10 years, family history of thyroid cancer) and ultrasound (irregular margins) risk factors, as well as the absence of enlarged cervical lymph nodes, mediastinal and lung invasion at CT scanning, DCT was initially not suspected in this case and a hemithyroidectomy was performed with a the preoperative diagnosis of a large toxic adenoma. In the presence of a size of more than 4 cm and an intense internal vascularisation (and calcifications in the nodule), an initial total thyroidectomy could have been justified, as suggested by Deluca et al. [26]. On the other hand, a family history of multinodular goitre and the absence of cervical and lung invasion was assessed as a reassuring feature in our case, favouring a left hemithyroidectomy. We first treated our patient with methimazole, as patients with overt hyperthyroidism due to a hyperfunctioning nodule should be euthyroid prior to the surgical procedure [25]. In all reported cases, euthyroidism was observed relatively quickly after medical treatment, as observed in our case.

Somatic mutations of the *TSHR* and *GNAS1* gene have been detected in adolescents presenting with autonomous functioning benign thyroid nodule as well as thyroid carcinoma with or without associated hyperthyroidism [8, 14, 22]. In one study, in 17 of 29 of benign hyperfunctioning nodules somatic TSHR mutations were found, while only one of the 4 studied DCT a mutation was found. Surgical specimen examination in our case was negative for *BRAF* and *NRAS* mutations and *RET/PTC* rearrangements. Increased malignancy rate of hyperfunctioning nodules was found not to be associated with *BRAF*, *NRAS* mutations and *PAX8/PPARG* and *RET/PTC* rearrangements [22].

## Conclusion

In conclusion, our case illustrates the difficulty to accurately determine the risk of DTC in hyperthyroid adolescents presenting with a toxic nodule and provides histological evidence that a large thyroid carcinoma can be composed of both a follicular and papillary carcinoma.

## Data Availability

Data sharing is not applicable to this article as no datasets were generated or analysed during the current study.

## Abbreviations

ATA: American Thyroid Association
CT: Computed Tomography
DTC: Differentiated Thyroid Cancer
FT3: Free Triiodothyonine
FT4: Free Thyroxine
TSH: Thyroid Stimulating Hormone

## Footnotes

1 Reference rate: FT4 12.0–22.0 pmol/L, thyroglobulin concentration 0.2–70.0 μg/L, TSH 0.70–6.4 mU/L
